# Rapid Detection and Isolation of *Escherichia coli* O104:H4 from Milk Using Monoclonal Antibody-coated Magnetic Beads

**DOI:** 10.3389/fmicb.2016.00942

**Published:** 2016-06-15

**Authors:** Mirella Luciani, Tiziana Di Febo, Katiuscia Zilli, Elisabetta Di Giannatale, Gisella Armillotta, Laura Manna, Fabio Minelli, Manuela Tittarelli, Alfredo Caprioli

**Affiliations:** ^1^Istituto Zooprofilattico Sperimentale dell’Abruzzo e del Molise “G. Caporale”, TeramoItaly; ^2^EU Reference Laboratory for E. coli, Department of Veterinary Public Health and Food Safety, Istituto Superiore di Sanità, RomeItaly

**Keywords:** detection, *Escherichia coli* O104:H4, immuno-magnetic separation, milk, monoclonal antibodies

## Abstract

Monoclonal antibodies (MAbs) specific for the lipopolysaccharide (LPS) of *Escherichia coli* O104:H4 were produced by fusion of Sp2/O-Ag-14 mouse myeloma cells with spleen cells of Balb/c mice, immunized with heat-inactivated and sonicated *E. coli* O104:H4 bacterial cells. Four MAbs specific for the *E. coli* O104:H4 LPS (1E6G6, 1F4C9, 3G6G7, and 4G10D2) were characterized and evaluated for the use in a method for the detection of *E. coli* O104:H4 in milk samples that involves antibody conjugation to magnetic microbeads to reduce time and increase the efficiency of isolation. MAb 1E6G6 was selected and coupled to microbeads, then used for immuno-magnetic separation (IMS); the efficiency of the IMS method for *E. coli* O104:H4 isolation from milk was evaluated and compared to that of the EU RL VTEC conventional culture-based isolation procedure. Milk suspensions also containing other pathogenic bacteria that could potentially be found in milk (*Campylobacter jejuni, Listeria monocytogenes*, and *Staphylococcus aureus*) were also tested to evaluate the specificity of MAb-coated beads. Beads coated with MAb 1E6G6 showed a good ability to capture the *E. coli* O104:H4, even in milk samples contaminated with other bacteria, with a higher number of *E. coli* O104:H4 CFU reisolated in comparison with the official method (121 and 41 CFU, respectively, at 10^3^ *E. coli* O104:H4 initial load; 19 and 6 CFU, respectively, at 10^2^ *E. coli* O104:H4 initial load; 1 and 0 CFU, respectively, at 10^1^ *E. coli* O104:H4 initial load). The specificity was 100%.

## Introduction

*Escherichia coli* (Enterobacteriaceae) is a Gram-negative, facultative anaerobic bacterium that is commonly found in the lower intestinal tract of healthy animals and humans. However, several *E. coli* strains have acquired virulence traits that allow them to cause disease in humans and animals. At least six categories of pathogenic *E. coli* able to affect the human gut have been described: Shiga-toxin-producing *E. coli*, also called verocytotoxin-producing *E. coli* (STEC or VTEC), of which enterohaemorrhagic *E. coli* (EHEC) are a highly pathogenic sub-group causing bloody diarrhea and the hemolytic uremic syndrome (HUS), characterized by severe acute renal failure, thrombocytopenia and micro-angiopathic haemolytic anemia (European Centre for Disease Prevention and Control [ECDC] and European Food Safety Authority [EFSA], 2011); enteropathogenic *E. coli* (EPEC); enterotoxigenic *E. coli* (ETEC); enteroaggregative *E. coli* (EAggEC); enteroinvasive *E. coli* (EIEC), and attaching and effacing *E. coli* (A/EEC) (European Centre for Disease Prevention and Control [ECDC] and European Food Safety Authority [EFSA], 2011; Farrokh et al., 2013).

Prior to 2011, STEC serogroup O104 was not considered as a major STEC serogroup, although it had been associated with an outbreak of diarrhea in the US and with sporadic cases in European countries and Korea (European Centre for Disease Prevention and Control [ECDC] and European Food Safety Authority [EFSA], 2011; Baranzoni et al., 2014).

The concern about this serogroup increased in May-July 2011, with the occurrence of two outbreaks of bloody diarrhea and HUS in Europe: one in Germany (around 4000 cases of bloody diarrhea, 850 cases of HUS and 50 deaths), and a much smaller outbreak in southwest France (15 cases of bloody diarrhea, 9 of which progressed to HUS). Both outbreaks were caused by a STEC strain belonging to serotype O104:H4 and linked to the consumption of contaminated sprouts from fenugreek seeds (Grad et al., 2012; Baranzoni et al., 2014). The genetic analysis of the outbreak strain revealed that it carried virulence genes associated with both STEC and EaggEC (Bielaszewska et al., 2011; Scheutz et al., 2011; Baranzoni et al., 2014); in addition, all isolates also expressed the phenotypes that define STEC and EaggEC, specifically production of Shiga-toxin 2 (Stx2) and the aggregative adherence pattern on intestinal epithelial cells, and were resistant to all penicillins and cephalosporins and to co-trimoxazole (trimethoprim-sulfamethoxazole). The specific combination of the higher adherence to intestinal cells, physical survival, Stx2 production and antibiotic resistance, shows the high genomic plasticity of *E. coli* O104:H4 and could explain the high virulence of the epidemic strain (Bielaszewska et al., 2011; Scheutz et al., 2011).

The severity of the oubreaks caused by this foodborne pathogen highlights the need for sensitive screening methods allowing its rapid identification and isolation from food matrices, as sprouts, milk and meat.

Raw cow’s and goat’s milk provides a potential growth medium for bacteria and its consumption has been frequently associated with STEC infections in Europe, USA and Canada. Most of these cases were associated with STEC O157, although other serotypes or serogroups, including O22:H8, O110:H^-^, O80:H^-^, and O145 have been identified as causative agents. Consumption of contaminated soft and semi-soft cheeses has also been implicated in outbreaks: *E. coli* O157:H7 was linked to the majority of cases, but O27:H20, O103, O26, O145, O119:B14, O27:H20, and O104:H21 have also been implicated (Centers for Diseases Control and Prevention [CDC], (1995); Farrokh et al., 2013). Generally, there are two suggested routes by which potentially pathogenic STEC can contaminate raw milk: rare sub-clinical mastitis causing STEC excretion from the udder and contamination during the milking process, when teats are soiled with feces. STEC could also potentially persist if milking equipment is not adequately cleaned. Contamination of dairy products (cheeses, cream, ice-cream, yogurt and butter) is commonly due to the use of raw/unpasteurized milk, to defective pasteurization of milk and/or post processing contamination (Farrokh et al., 2013).

The aim of this work was the development of an immuno-magnetic separation (IMS) method based on the use of beads coated with monoclonal antibodies (MAbs) specific for the lipopolysaccharide (LPS) of *E. coli* O104:H4 for the rapid and efficient isolation of *E. coli* O104:H4 from milk samples.

## Materials and Methods

### Bacterial Strains

The *E. coli* O104:H4 strain used for the production and the screening of MAbs and for the immunomagnetic capture was isolated from an Italian child with HUS in 2009 (Scavia et al., 2011). Other five enteroaggregative *E. coli* O104 strains were used to test the MAb 1E6G6. These included two VT2-positive strains and one VT-negative strain kindly provided by the Statens Seruminstitut (Copenhagen, Denmark), a VT2-positive strain kindly provided by the Robert Koch Institute (Berlin, Germany), and another VT2-positive strain kindly provided by the Hopital Robert Debre (Paris, France). The *E. coli* O157, *E. coli* O26, *E. coli* O103, *E. coli* O111, and *E. coli* O145 strains used for MAbs characterization, were part of the culture collection of the European Union Reference Laboratory for *E. coli* (EU-RL-ISS). *Campylobacter jejuni* ATCC 33291, *Listeria monocytogenes* ATCC 7644, and *Staphylococcus aureus* ATCC 6538 used to evaluate the specificity of MAbs, were obtained from ATCC Bacterial Collection LGC.

### Preparation of *E. coli* Antigens for MAbs Production and Characterization

*Escherichia coli* O104:H4, *E. coli* O157, *E. coli* O26, *E. coli* O103, *E. coli* O111, and *E. coli* O145 were grown in Brain Heart Infusion Broth (BHI) (Oxoid, Basingstoke, Hampshire, UK) at 37°C for 14–16 h, inactivated at 100°C for 1 h and centrifuged at 5,500 *g* for 30 min. The pellet was washed for three times with 0.01 M phosphate buffered saline, pH 7.2 (PBS), resuspended in PBS and stored at –20°C. One aliquot of the *E. coli* O104:H4 suspension was sonicated in ice bath for two cycles of 2.5 min each with an interval of 5 min and immediately used for mice immunization.

The protein concentration of antigen preparations was determined using the BCA Protein Assay Kit (Pierce Rockford, IL, USA).

### Preparation of the *E. coli* O104 Lipopolysaccharide

The *E. coli* O104 LPS was extracted by the hot phenol–water method (Westphal and Jann, 1965). The LPS was precipitated with 3 M sodium-acetate and absolute ethanol, ultracentrifuged, resuspended in deionized water and stored at –80°C until use. The quantity of LPS was determined by the 2-keto-3-deoxyoctonate (KDO) assay (Karkharis et al., 1978).

### Immunization of Mice

For MAbs production, 6/8 week-old Balb/c mice were inoculated with heat-inactivated and sonicated *E. coli* O104:H4 preparations. Animal experimentation was carried out in compliance with Italian national law (Decreto legislativo 27 Gennaio, 1992, n. 116) implementing Directive 86/609/EEC of the Council of the European Communities on the protection of animals used for experimental and other scientific purposes (European Commission [EC], 1986). The protocol was approved by the Italian Ministry of Health with number 5146 of 26.04.2012. The whole antigen, diluted to a protein concentration of 100 μg/ml, was emulsified with complete Freund adjuvant (Sigma, St. Louis, MO, USA) and administered intraperitoneally; 14 days later a second immunization was performed using the same concentration of antigen emulsified with incomplete Freund adjuvant (Sigma). Subsequently, on days 28 and 56, 100 μg/ml of antigen diluted in PBS was given (intravenous booster). Three days later, the mice were sacrificed, the spleen collected and splenocytes subjected to cell fusion with murine myeloma cells Sp2/O-Ag-14 (ATCC CRL-1581^TM^).

### Characterization of Monoclonal Antibodies vs. *E. coli* O104:H4

The antibody-secreting hybridomas, cultured in Dulbecco’s Modified Eagle’s Medium (DMEM) containing 20% bovine fetal serum, 2 mM L-glutamine, 100X Penicillin–Streptomycin–Amphotericin, 50 mg/ml gentamicin and 50X HAT, were screened by i-ELISA. Briefly, 96-well microplates (PolySorp, Nunc Brand Products, Roskilde, DK) were coated with 10 μg/ml of *E. coli* O104 LPS and of the bacterial whole antigens (*E. coli* O104:H4, *E. coli* O157, *E. coli* O26, *E. coli* O103, *E. coli* O111, *E. coli* O145) diluted in 0.05 M carbonate-bicarbonate buffer, pH 9.6, and incubated overnight at 4°C. After washing and blocking with 1% yeast extract in PBS containing 0.05% Tween 20 (PBST) at 37°C for 1 h, 100 μl/well of MAbs supernatants were added and incubated for 1 h at 37°C. As secondary antibody, ECL anti-mouse IgG conjugated with horseradish peroxidase (GE Healthcare, Little Chalfont, Buckinghamshire, UK) was used; the 3,3′,5,5′-tetramethylbenzidine (TMB, Sigma) was adopted as chromogenic substrate. Microplate reading was performed with a biophotometer (Bio-Rad, Hercules, CA, USA) at a wavelength of 450 nm. Clones that showed optical densities (OD_450_) greater than or equal to 3 times the OD_450_ of the negative control (serum of a naive Balb/c mouse) were considered as positives and were cloned by the limiting dilution method (Luciani et al., 2006). MAbs were produced *in vitro* on a large scale by means of serial cultures of hybridomas and collection of the supernatants and were isotyped using the Mouse-Typer Isotyping Panel (Bio-Rad).

For immunoblotting analysis, MAbs with IgG and IgM isotype were purified on affinity chromatography using, respectively, a HiTrap rProtein A FF column (GE Healthcare, Uppsala, SW) and a HiTrap IgM Purification HP column (GE Healthcare), according to the manufacturer’s instructions.

Purified MAbs were concentrated with 100 kDa cut-off centrifugal filters (Millipore, Billerica, MA, USA) and resuspended in PBS. The protein concentration of purified MAbs was determined by spectrophotometry (Absorbance at 280 nm/IgG molar extinction coefficient).

The heat-treated *E. coli* O104:H4 suspensions (2.5 μg/well) were subjected to SDS-PAGE separation at 200 V with NuPAGE 4–12% Bis-Tris Gels Mini (Novex, Life Technologies, Carlsbad, CA, USA) and transferred onto nitrocellulose membrane with iBlot Dry Blotting System (Life Technologies). After blocking with 5% skimmed milk (Fluka Analytical, Sigma-Aldrich) in PBST for 2 h at room temperature (RT), membrane strips were incubated overnight at 4°C with the purified MAbs. The detection of immune complexes was performed using the ECL anti-mouse IgG HRP-conjugated (GE Healthcare) and a chemiluminescent substrate (ECL Select Western Blotting Detection Reagent, GE Healthcare). The analysis of the results was performed using the Chemidoc MP (Bio-Rad) and the Quantity One Quantitation Software version 4.3 (Bio-Rad).

### Magnetic Beads Coupling Procedure

Purified MAbs were coupled to Dynabeads M-450 Tosylactivated (Life Technologies). Twenty-five micro litre of dynabeads were first washed with 0.1 M sodium phosphate buffer, pH 7.4, and then incubated with 5 μg of each purified MAb at 37°C for 24 h using the Dynabeads MX4 Mixer (Life Technologies). Supernatant was removed and dynabeads were resuspended in 0.1 M Tris containing 0.1% BSA (pH 8.5) and incubated for 4 h at 37°C with gentle and continuous agitation. Supernatant was discarded and activated dynabeads were resuspended in PBS containing 0.1% BSA, 2 mM EDTA (pH 7.4), and stored at 4°C until use.

Non-coated dynabeads were also prepared using the same protocol, but without the incubation with the MAbs (non-coated-beads control as blank).

### Preparation of Bacterial Strains for Immuno-Magnetic Separation

*Escherichia coli* O104:H4 was grown in Tryptic Soy Broth (TSB) (Becton Dickinson, Franklin Lakes, NJ, USA) for 24 h at 37°C, and then the cultures were streaked onto Tryptic Soy Agar (TSA) (Becton Dickinson) plates and incubated for 24 h in aerobic atmosphere at 37°C. A well separated colony from the plate was transferred into 10 ml of TSB and incubated at 37°C in order to obtain a bacterial suspension with a turbidity between 2 and 3 McFarland standards. The number of cells in the bacterial suspension was also estimated by reading the absorbance at 600 nm (OD_600_). The final concentration of bacterial suspension was 10^11^ CFU. The culture was decimally diluted in sterile PBS (until 10^-11^); the number of bacteria in each diluted suspension was confirmed by plating onto MacConkey Agar at 37°C for 24 h.

*Campylobacter jejuni, L. monocytogenes*, and *S. aureus* (pool of contaminating bacteria) were grown, respectively, on Karmali Agar, Aloe Agar, and TSA Agar. For each strain, a bacterial suspension in PBS with a concentration of 0.8 McFarland (about 10^8^ CFU) was prepared.

### Immuno-Magnetic Separation and Evaluation of MAb-Coated Beads Specificity

The specificity of MAb-coated beads was first evaluated using PBS suspensions, prepared as described above, containing decreasing concentration of *E. coli* O104:H4 from 10^6^ CFU to 10^1^ CFU. Tests were repeated using *E. coli* O104:H4 PBS suspensions containing *L. monocytogenes*, *C. jejuni* and *S. aureus* at the concentration of 10^3^ CFU each.

One ml of each PBS-diluted bacterial suspension was added with 25 μl of MAb-activated dynabeads and incubated at 4°C for 30 min with gentle and continuous agitation. Then, dynabeads were washed four times with PBS containing 0.1% BSA and 2 mM EDTA, (pH 7.4), resuspended in 100 μl of PBS, plated onto MacConkey Agar plates and incubated at 37°C for 24 h. The same procedure was repeated using non-coated beads to ensure the absence of aspecific interactions between the non-coated beads and bacteria.

### Inoculation and Enrichment of Milk and *E. coli* O104:H4 Detection from Artificially Contaminated Milk Samples

Six aliquots (9 ml each) of bovine raw milk were artificially contaminated with 1 ml of each one of the six bacterial suspensions previously prepared (*E. coli* O104:H4 concentration from 10^6^ CFU to 10^1^ CFU). One ml of each *E. coli* O104:H4 contaminated milk sample was incubated with MAb-coated and non-coated beads as described above. In the same time, one ml aliquot of each milk sample was analyzed using the EU RL VTEC official method, which include an overnight enrichment step. Aliquots (100 μl) of the IMS-treated samples and of the enrichment broth cultures prepared according to the EU RL VTEC method were plated onto MacConkey Agar plates. After incubation for 24 h at 37°C in aerobic condition, the colonies obtained with the EU RL VTEC method and with MAb-coated beads were enumerated.

To verify the absence of cross-reaction between MAbs and bacteria other than *E. coli* O104:H4, the same tests described above were repeated using four aliquots for each of the six cream milk samples artificially contaminated with the six bacterial suspensions at the concentration of *E. coli* O104:H4 from 10^6^ CFU to 10^1^ CFU and with each species of contaminating bacteria at the concentration of 10^3^ CFU. Each of four aliquots of the six milk suspensions were, respectively, plated onto MacConkey Agar, onto Blood Agar for 24 h at 37°C in aerobic condition for *L. monocytogenes* and *S. aureus* isolation, and Karmali Agar for 48 h at 42°C in microaerobic conditions for *C. jejuni* isolation.

### DNA Extraction and PCR for Strains Typing

*Escherichia coli* O104:H4 DNA extraction was performed using the InstaGene Matrix (Bio-Rad) according to manufacturer’s instructions. Primers used for the PCR assay were specific for the *rfb_O104_* gene, encoding the O-antigen specific for *E. coli* O104 (O104*rfb*O-f 5′ TGAACTGATTTTTAGGATGG 3′; O104*rfb*O-r 5′ AGAACCTCACTCAAATTATG 3′, amplicon size 351 bp) (Bielaszewska et al., 2011). A 50 μl PCR mixture contained: 25 μl 2× TopTaq Master Mix (Qiagen, Venlo, NL) (1.25 units of TopTaq DNA Polymerase, 1× PCR buffer, dNTPs concentration 0.2 mM each, 1.5 mM MgCl_2_), 1 μl of each primer at the concentration of 0.2 μM, 18 μl of Nuclease-Free Water and 5 μl of template DNA. Amplifications were performed using the GeneAmp^®^ PCR System 9700 (Applied Biosystems, Life Technologies) and the following temperature cycling conditions: initial denaturation at 95°C for 5 min, followed by 35 cycles of denaturation at 95°C for 30 seconds, annealing at 55°C for 1 min, extension at 72°C for 1 min and final extension at 72°C for 7 min. The PCR products were resolved by electrophoresis on a 1,5% agarose gel.

### Statistical Analysis

In order to determine differences between the EU RL VTEC conventional culture-based isolation procedure and the IMS method described in this paper, the Wilcoxon test for dependent samples was applied (confidence interval = 95%).

## Results

### Characterization of Monoclonal Antibodies vs. the LPS of *E. coli* O104:H4

**FIGURE 1:**
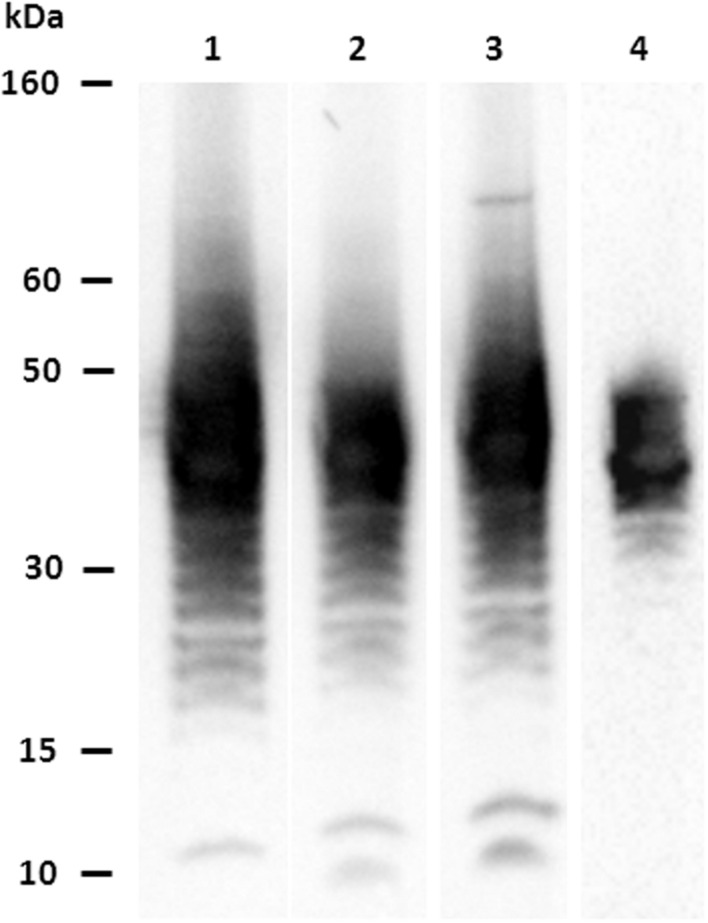
**Characterization of MAbs vs. *Escherichia coli* O104:H4: western blotting results.** Lane 1: MAb 1E6G6; lane 2: MAb 1F4C9; lane 3: MAb 3G6G7; lane 4: MAb 4G10D2.

A total of 28 hybridomas were positive against the *E. coli* O104:H4 whole heat-treated antigen in indirect ELISA. Four MAbs (1E6G6, 1F4C9, 3G6G7, and 4G10D2) were found positive for both the *E. coli* O104:H4 LPS and whole antigen, and negative against the other O serogroups tested. MAbs isotypes are shown in **Table 1**. Characterization by western blotting revealed that the four selected MAbs reacted with the LPS of *E. coli* O104:H4; **Figure 1** shows the ladder-like pattern of LPS bands with molecular weights between 60 and 15 kDa recognized by the MAbs. One of the four selected MAbs, MAb 1E6G6, was tested by slide agglutination against five additional *E. coli* O104:H4 strains with positive results and subsequently conjugated to magnetic microbeads, to be used for a specific IMS of *E. coli* O104:H4 from milk samples.

**Table 1:** Monoclonal antibodies isotypes.

Clone	Isotype
1E6G6	IgG1 anti κ
1F4C9	IgG3 anti κ
3G6G7	IgG1 anti κ
4G10D2	IgG3 anti κ

### Immuno-Magnetic Separation and Evaluation of MAb-Coated Beads Specificity

The results obtained from *E. coli* O104:H4 re-isolation from PBS pure bacterial suspensions are shown in **Table 2**. At *E. coli* concentrations higher than or equal to 10^4^, the number of CFU re-isolated was higher than 200. At the concentrations of 10^3^ and 10^2^ CFU, 76 and 3 CFU, respectively, were re-isolated following the IMS.

**Table 2:** Evaluation of MAb-coated beads specificity using *Escherichia coli* O104:H4 PBS pure suspensions.

*E. coli* O104:H4 in the initial suspension (CFU number)	*E. coli* O104:H4 re-isolated after immuno-magnetic separation (IMS) (CFU number)
10^6^	>200
10^5^	>200
10^4^	>200
10^3^	76
10^2^	3
10^1^	0

When the *E. coli* O104:H4 PBS suspensions containing other bacteria (*L. monocytogenes*, *C. jejuni* and *S. aureus*) were used, more than 200 *E. coli* O104:H4 CFU were re-isolated from suspensions with *E. coli* concentrations higher than or equal to 10^3^; 19 and 2 CFU were re-isolated from the 10^2^ and 10^1^ CFU *E. coli* O104:H4 suspensions, respectively, (**Table 3**). None of the contaminating bacteria were captured by the MAb-coated beads. No aspecific binding between the non-coated beads and the four bacterial suspensions used in the experiments was observed.

**Table 3:** Evaluation of MAb-coated beads specificity using *E. coli* O104:H4 PBS suspensions containing contaminating bacteria (*Listeria monocytogenes*, *Campylobacter jejuni*, and *Staphylococcus aureus*).

*E. coli* O104:H4 in the initial suspension (CFU number)	Contaminating bacteria in the initial suspension (CFU number)	*E. coli* O104:H4 re-isolated after IMS (CFU number)	Contaminating bacteria re-isolated after IMS (CFU number)
10^6^	10^3^	>200	0
10^5^	10^3^	>200	0
10^4^	10^3^	>200	0
10^3^	10^3^	>200	0
10^2^	10^3^	19	0
10^1^	10^3^	2	0

### *E. coli* O104:H4 Detection in Artificially Contaminated Milk Samples

**Table 4** shows the results of the *E. coli* O104:H4 isolation from milk samples contaminated with scalar dilutions (from 10^6^ to 10^1^ CFU) of *E. coli* O104:H4. With the Dynabeads method, a higher number of *E. coli* O104:H4 CFU was isolated with respect to the EU RL method. Similar results were obtained with milk samples contaminated with *E. coli* O104:H4 and with other bacteria (*L. monocytogenes*, *C. jejuni* and *S. aureus*) (**Table 5**). The p-value obtained using the Wilcoxon test for dependent samples was 0.0517 with a confidence interval of 95%.

**Table 4:** *Escherichia coli* O104:H4 isolation from artificially contaminated milk samples in the absence of contaminating bacteria: comparison between the EU RL VTEC official method and the *E. coli* O104 Dynabeads method.

*E. coli* O104:H4 in the initial suspension (CFU number)	*E. coli* O104:H4 isolated with the EU RL method (CFU number)	*E. coli* O104:H4 isolated with the O104 Dynabeads method (CFU number)
10^6^	>200	>200
10^5^	159	>200
10^4^	117	>200
10^3^	43	>200
10^2^	8	27
10^1^	0	4

**Table 5:** *Escherichia coli* O104:H4 detection from artificially contaminated milk samples in the presence of contaminating bacteria (*L. monocytogenes*, *C. jejuni*, and *S. aureus*): comparison between the EU RL VTEC official method and the *E. coli* O104 Dynabeads method.

*E. coli* O104:H4 in the initial suspension (CFU number)	Contaminating bacteria in the initial suspension (CFU number)	*E. coli* O104:H4 isolated with EU RL method (CFU number)	*E. coli* O104:H4 isolated with the *E. coli* O104 Dynabeads method (CFU number)	*S. aureus* isolated with the *E. coli* O104 Dynabeads method (CFU number on Blood Agar plate)	*L. monocytogenes* isolated with the *E. coli* O104 Dynabeads method (CFU number on Blood Agar plate)	*C. jejuni* isolated with the *E. coli* O104 Dynabeads method (CFU number on Karmali Agar plate)
10^6^	10^3^	>200	>200	0	0	0
10^5^	10^3^	>200	>200	0	0	0
10^4^	10^3^	137	>200	0	0	0
10^3^	10^3^	41	121	0	0	0
10^2^	10^3^	6	19	0	0	0
10^1^	10^3^	0	1	0	0	0

**FIGURE 2:**
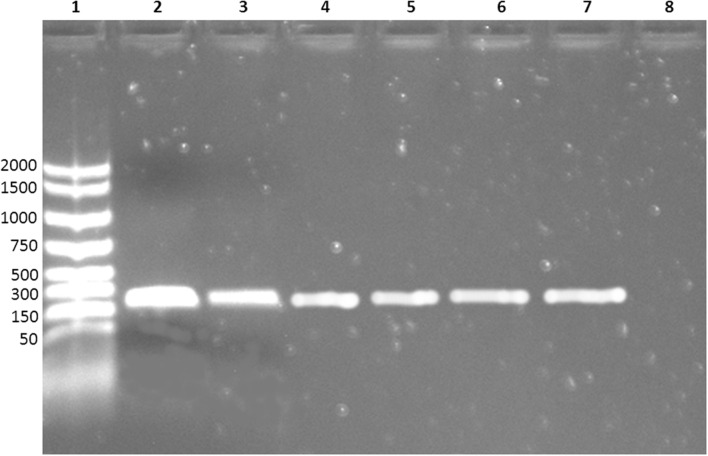
**Electrophoresis of the PCR products.** Lane 1: molecular weight marker; lane 2–6: *E. coli* O104:H4 captured by MAb-coated magnetic beads; lane 7: positive control (DNA extracted from *E. coli* O104:H4 ISS-certified); lane 8: negative control (nuclease-free water).

Moreover, none of the other contaminating bacteria were captured by MAb-coated beads, as shown by the absence of growth in the specific media. MAb-coated beads isolates were further confirmed by PCR: all the bacteria captured by coated beads were *E. coli* O104:H4 (**Figure 2**).

## Discussion and Conclusion

Shiga toxin–producing *Escherichia coli* strains are important foodborne pathogens that have been responsible for numerous outbreaks of hemorrhagic colitis and HUS worldwide. *E. coli* O157:H7 is the most frequently implicated in human disease, although infections can be caused by other serotypes or serogroups, as *E. coli* O104:H4. Consequently it is necessary to have the availability of rapid, sensitive and specific methods for food control.

In this study, MAbs vs. *E. coli* O104:H4 were produced andcharacterized. The efficiency of the *E. coli* O104:H4 isolation from milk samples, using Dynabeads activated with a selected MAb (1E6G6) specific for *E. coli* O104:H4 LPS, was evaluated and compared with that of the EU RL VTEC isolation procedure (European Reference Laboratory for *E.coli* [EU-RL VTEC], 2011). The specificity of the adsorbed antibodies was also evaluated by preparing *E. coli* O104:H4 suspensions containing other pathogenic bacteria that could potentially be found in milk, such as *Campylobacter jejuni, Listeria monocytogenes*, and *Staphylococcus aureus* (Farrokh et al., 2013).

The use of MAbs, instead of polyclonal ones (PAbs), could be a valid alternative to improve the specificity of pathogen detection. PAbs may have a lack of homogeneity between lots, due to the variability of the animal immune response. They may also show cross-reactions with related pathogens. Conversely, MAbs are highly specific reagents, easily purified and with batch to batch homogeneity.

Immuno-magnetic separation is a rapid and sensitive method that permit the revelation of low concentrations of pathogens in contaminated matrices. The magnetic beads method has already been applied for the detection of *E. coli* O157:H7 (Weagant et al., 2011) and *E. coli* O104 (Baranzoni et al., 2014) from sprouts with good results. In particular, Baranzoni et al. (2014) used commercially available *E. coli* IMS beads in association with optimized enrichment conditions of sprouts samples. The results indicated that the IMS enrichment enhanced the ability to detect *E. coli* O104 and reduced the interference of background microflora. In our work, we used O104-specific magnetic beads prepared in-house to detect this pathogen in artificially contaminated milk samples, skipping the use of a previous enrichment step. The procedure showed an analytical sensitivity higher than that of the isolation procedure included in the official EURL method for *E. coli* O104. Even if the statistic analysis gave a value slightly higher than the expected one, numeric data show that the immunomagnetic method is more sensitive, particularly at low concentrations of bacteria.

The MAb-coated beads showed a good capability to capture *E. coli* O104:H4 in artificially contaminated milk samples, even in the presence of other contaminating bacteria, such as *C. jejuni*, *L. monocytogenes*, and *S. aureus*, with an increase in the number of CFU re-isolated with respect to the current official method (>200 and 137 CFU, respectively, at 10^4^ *E. coli* O104:H4 initial load; 121 and 41 CFU, respectively, at 10^3^ *E. coli* O104:H4 initial load; 19 and 6 CFU, respectively, at 10^2^ *E. coli* O104:H4 initial load and 1 and 0 CFU, respectively, at 10^1^ *E. coli* O104:H4 initial load). The Dynabeads method also showed a good analytical specificity, since no contaminating bacteria were captured by the MAb-coated beads in both PBS bacterial suspensions and contaminated milk samples and no aspecific reactions were obtained with non-coated beads. MAb 1E6G6 was able to retain its antigen-binding activity even in the presence of a fat matrix such as full cream milk. Moreover, the direct application of the O104 IMS to a liquid matrix such as milk, skipping the broth enrichment step that requires an overnight incubation period, allowed a more rapid isolation of *E. coli* O104, with respect to the current official method.

Thus, magnetic beads linked with the MAb 1E6G6 against *E. coli* O104:H4 could be a useful tool for improving the detection of this *E. coli* serogroup in milk samples. Further studies are needed to evaluate the performances of MAb 1E6G6 coated-beads when matrices other than from milk are tested.

## Author Contributions

ML, TD, and AC designed the study; ML, TD, GA, LM, FM, and MT produced, characterized and purified monoclonal antibodies; AC, FM, KZ, and ED performed microbiological analysis; ML coordinated animal management; ML, TD, and KZ drafted the manuscript; AC, ED, and MT revised the paper critically. All authors read and approved the final version of the manuscript.

## Conflict of Interest Statement

The authors declare that the research was conducted in the absence of any commercial or financial relationships that could be construed as a potential conflict of interest.

## References

[B1] Baranzoni G. M. Fratamico P. M. Rubio F. Glaze T. Bagi L. K. Albonetti S. (2014). Detection and isolation of Shiga toxin-producing *Escherichia coli* (STEC) O104 from sprouts. *Int. J. Food Microbiol.* 173 99–104. 10.1016/j.ijfoodmicro.2013.12.020 24413585

[B2] Bielaszewska M. Mellmann A. Zhang W. Köck R. Fruth A. Bauwens A. (2011). Characterisation of the *Escherichia coli* strain associated with an outbreak of haemolytic uraemic syndrome in Germany, 2011: a microbiological study. *Lancet Infect. Dis.* 11 671–676. 10.1016/S1473-3099(11)70165-7 21703928

[B3] Centers for Diseases Control and Prevention [CDC] (1995). Outbreak of acute gastroenteritis attributable to *Escherichia coli* serotype O104:H21 – Helena, Montana, 1994. *MMWR Morb. Mortal. Wkly. Rep.* 44 501–503. 7596334

[B4] Decreto legislativo 27 Gennaio (1992). n. 116. Attuazione della direttiva n. 86/609/CEE in materia di protezione degli animali utilizzati a fini sperimentali o ad altri fini scientifici. *Official J.* 40 1–32.

[B5] European Centre for Disease Prevention and Control [ECDC] and European Food Safety Authority [EFSA] (2011). *Shiga Toxin/Verotoxin-Producing Escherichia coli in Humans, Food and Animals in the EU/EEA, with Special Reference to the German Outbreak Strain STEC O104.* ECDC/EFSA Joint Technical Report (Stockolm: ECDC), 1–18.

[B6] European Commission [EC] (1986). Council Directive of 24 November 1986 on the approximation of laws, regulations and administrative provisions of the Member States regarding the protection of animals used for experimental and other scientific purposes (86/609/EEC). *Official J.* L358 1–28. 20397315

[B7] European Reference Laboratory for *E.coli* [EU-RL VTEC] (2011). *Detection and Identification of Verocytotoxin-Producing Escherichia coli (VTEC) O104:H4 in food by Real Time PCR.* Rome: Istituto Superiore di Sanità.

[B8] Farrokh C. Jordan K. Auvray F. Glass K. Oppegaard H. Raynaud S. (2013). Review of Shiga-toxin-producing *Escherichia coli* (STEC) and their significance in dairy production. *Int. J. Food Microbiol.* 162 190–212. 10.1016/j.ijfoodmicro.2012.08.008 22939912

[B9] Grad Y. H. Lipsitch M. Feldgarden M. Arachchi H. M. Cerqueira G. C. Fitzgerald M. (2012). Genomic epidemiology of the *Escherichia coli* O104:H4 outbreaks in Europe, 2011. *Proc. Natl. Acad. Sci. U.S.A.* 109 3065–3070. 10.1073/pnas.1121491109 22315421 PMC3286951

[B10] Karkharis Y. D. Zeltner J. Y. Jackson J. J. Carlo D. J. (1978). A new and improved microassay to determine 2-keto-3-deoxyoctonate in lipopolysaccharide of gram-negative bacteria. *Anal. Biochem.* 85 595–601. 10.1016/0003-2697(78)90260-9 646115

[B11] Luciani M. Armillotta G. Magliulo M. Portanti O. Di Febo T. Di Giannatale E. (2006). Production and characterization of monoclonal antibodies specific for *Escherichia coli* O157:H7. *Vet. Ital.* 42 173–182. 20429059

[B12] Scavia G. Morabito S. Tozzoli R. Michelacci V. Marziano M. L. Minelli F. (2011). Similarity of Shiga toxin-producing *Escherichia coli* O104:H4 strains from Italy and Germany. *Emerg. Infect. Dis.* 17 1957–1958. 10.3201/eid1710.111072 22000382 PMC3310690

[B13] Scheutz F. Nielsen E. M. Frimodt-Møller J. Boisen N. Morabito S. Tozzoli R. (2011). Characteristics of the enteroaggregative Shiga toxin/verotoxin-producing *Escherichia coli* O104:H4 strain causing the outbreak of haemolytic uraemic syndrome in Germany, May to June 2011. *Euro Surveill.* 16 5–10. 10.2807/ese.16.24.19889-en 21699770

[B14] Weagant S. D. Jinneman K. C. Yoshitomi K. J. Zapata R. Fedio W. M. (2011). Optimization and evaluation of a modified enrichment procedure combined with immunomagnetic separation for detection of *E. coli* O157:H7 from artificially contaminated alfalfa sprouts. *Int. J. Food Microbiol.* 149 209–217. 10.1016/j.ijfoodmicro.2011.06.008 21784545

[B15] Westphal O. Jann K. (1965). Bacterial lipopolysaccharides, extraction with phenol–water and further application of the procedure. *Methods Carbohydr. Chem.* 5 83–91.

